# Valuation of Mortality Risk Attributable to Climate Change: Investigating the Effect of Survey Administration Modes on a VSL

**DOI:** 10.3390/ijerph9124760

**Published:** 2012-12-18

**Authors:** Milan Ščasný, Anna Alberini

**Affiliations:** 1 Charles University Prague, Environment Center, Jose Martiho 2, 162 00 Praha 6, Czech Republic; 2 University of Maryland, AREC, 2200 Symons Hall, College Park, MD 20742, USA; E-Mail: aalberini@arec.umd.edu; 3 Fondazione Eni Enrico Mattei, Isola di San Giorgio Maggiore, Venice 30124, Italy; 4 School of Biological Sciences and Institute for a Sustainable World, Queen’s University Belfast, Medical Biology Centre, 97 Lisburn Road, Belfast BT9 7BL, UK

**Keywords:** Value of Statistical Life (VSL), mortality risk, health benefit, climate change impacts, conjoint choice experiments, survey administration, Computer Assisted Personal Interviewing (CAPI), Computer Assisted Web Interviewing (CAPI), Czech Republic

## Abstract

The health impact attributable to climate change has been identified as one of the priority areas for impact assessment. The main goal of this paper is to estimate the monetary value of one key health effect, which is premature mortality. Specifically, our goal is to derive the value of a statistical life from people’s willingness to pay for avoiding the risk of dying in one post-transition country in Europe, *i.e.*, the Czech Republic. We carried out a series of conjoint choice experiments in order to value mortality risk reductions. We found the responses to the conjoint choice questions to be reasonable and consistent with the economic paradigm. The VSL is about EUR 2.4 million, and our estimate is comparable with the value of preventing a fatality as used in one of the integrated assessment models. To investigate whether carrying out the survey through the internet may violate the welfare estimate, we administered our questionnaire to two independent samples of respondents using two different modes of survey administration. The results show that the VSLs for the two groups of respondents are €2.25 and €2.55 million, and these figures are statistically indistinguishable. However, the key parameters of indirect utility between the two modes of survey administration are statistically different when specific subgroups of population, such as older respondents, are concerned. Based on this evidence, we conclude that properly designed and administered on-line surveys are a reliable method for administering questionnaires, even when the latter are cognitively challenging. However, attention should be paid to sampling and choice regarding the mode of survey administration if the preference of specific segments of the population is elicited.

## 1. Introduction

Impact on human health has been identified as one of the key effects of climate change. For instance, the PESETA research project that focuses on projecting the economic impacts of climate change on various sectors in Europe considers human health one of the priority areas for impact assessment [1]. The importance of health impacts from climate change is also emphasized by the World Health Organization [2] or the Intergovernmental Panel on Climate Change reports [3].

The IPCC 2007 WG2 report, Chapter 8, summarizes the main health impacts in five broad categories that include increased deaths, disease and injury due to heat waves, and extreme weather events; increased burden of diarrheal disease; altered spatial distribution of some infectious disease vectors; increased frequency of cardio-respiratory diseases due to higher concentrations of ground-level ozone related to climate change; and increases in malnutrition and consequent disorders, with implications for child growth and development [4].

These health impacts are usually quantified in terms of premature mortality or new cases of a certain illness. Alternatively, each health outcome can be expressed through one of the health impact indexes such as Disability Adjusted Life Years (DALY) or Quality Adjusted Life Years (QALY) that allow aggregation of all the adverse health effects and the expression of all of them through one health impact indicator. The effect on risk of dying can be expressed via a life-years-loss measure that basically recalculates all cases of premature mortality across all age groups, taking into consideration their life expectancies, into total years “lost” in a given population. If one of these indicators is then linked to involved costs, the cost-effectiveness of various policy designs or regulatory programs can be analysed. Because economic costs and health impacts are still expressed in quite different units, the costs and the health impacts cannot be summed up and the net benefit of a policy cannot be derived.

In order to derive the net benefit of a programme, the health impacts need to be monetized. Some like Trærup *et al.* in this volume [5], for instance, monetize DALYs through a value of life year (VOLY) when assessing the costs of cholera, such as that estimated, for example, in [6]. Deriving a monetary equivalent of the DALY/QALYs can provide a useful piece of information for a health impact assessment. However, this approach cannot be followed in a cost-benefit analysis either, due in particular to the incompatibility of the QALY/DALY measures with economic theory and welfare analysis.

Therefore, the only way how to include health impacts in a cost-benefit analysis in a proper way is to monetize each of the health outcomes, including mortality risks, with a corresponding monetary value based on a non-market valuation study. Then, the more monetized health impacts that are included, the more comprehensive the cost-benefit analysis that is performed.

Our main goal in this paper is to contribute to the literature on the costs of climate change, particularly on the cost of one specific impact category attributable to climate change, which is premature mortality. Specifically, our paper aims to derive the value of a statistical life from people’s willingness to pay for avoiding the risk of dying in one post-transition country in Europe—the Czech Republic. We also want to find whether the monetary values of premature deaths that have been used in assessing the social cost of carbon by means of the integrated assessment models can be justified.

Secondly, we want to investigate the potential for a wider use of alternative survey applications in order to derive similar welfare estimates for health impacts attributable to climate change in some other countries or regions. It is simply matter of fact that despite the growing literature on the valuation of mortality risks in the USA and some European countries, there is still little empirical evidence on how the monetary value for changes in the risk of dying might be in other regions. Although the method to derive the value of a statistical life is quite well established and accepted, the rather costly data collection process presents one of the main obstacles for wider survey applications around the world.

One way of overcoming this obstacle—at least in some regions—is to carry out the survey through the internet. The internet also offers an opportunity for performing a wide range of experimental treatments in stated preference research and, as a consequence, non-market valuation researchers have lately increasingly turned to on-line surveys, e.g., [7,8,9,10,11,12]. One of the specific purposes of this paper is to investigate whether this mode of administration produces the same results as computer-assisted personal interviews when the topic of the survey is complex and cognitively demanding, as is the case with mortality risk reductions.

On the other hand, there is a concern whether an internet-based survey administration may possibly bias responses and thus welfare estimates due to the representation of the internet sample or due to the different method of the survey administration itself. To shed light on the possible effect of the survey method of administration, Lindhejm and Navrud [13] reviewed the non-market valuation literature and found that the majority of welfare estimates did not actually differ for the internet and other modes of survey administration, and in a few cases the welfare estimates are somewhat lower for the internet surveys. Should the internet mode of administration violate the impact estimate, we would administered the questionnaire to two independent samples of Czech respondents using two different modes of survey administration. Specifically, we carried out a series of discrete choice experiments in order to value mortality risk reductions through interviews conducted in person and on-line using an e-panel of respondents.

In our survey, each person was asked to examine several pairs of hypothetical risk reduction profiles and each profile was defined by four attributes: the size of the risk reduction, whether the risk reduction is effective only for this decade or is repeated for several decades, whether it starts right away or is delayed, and the cost, to be paid annually for each of 10 years. Respondents are then asked to indicate their most preferred alternative out of risk reduction profile A, risk reduction profile B, and the status quo. We administered this questionnaire to 2,400 individuals using computer-assisted personal interviews at their home, and collected 800 more interviews from a comparable on-line sample in the same country.

The results show that two groups of respondents hold virtually the same marginal utilities of a unit risk reduction, income, and discount future risk reductions at the same rate. The VSLs are €2.25 and €2.55 million (PPP euro) in the CAWI and CAPI samples, and these figures are statistically indistinguishable. However, the equality of VSLs does not hold if we compare VSL estimates for specific subgroups, e.g. younger and older than 50, in which sample characteristics significantly differ. Based on this evidence, we conclude that even with complex, cognitively challenging concepts, on-line surveys produce reasonable and reliable results.

The remainder of the paper is organized as follows: Section 2 summarizes the main findings on the mortality effect of climate change, while the next section introduces the concept of valuation of mortality risks. Section 4 introduces our discrete choice experiments, sampling plan and survey implementation. Section 5 describes the data and following section summarizes the main results. The final section concludes the article.

## 2. Mortality Effect of Climate Change

Climate change may involve a range of complex inter-linkages with health. Direct impacts may include health outcomes related to temperature or changes in rainfall patterns, or impacts due to extreme weather events. Other impacts may follow more indirect pathways and are associated with water and food-borne diseases, or vector and rodent-borne diseases. Health impacts may also involve shortages in food or water supply [1,4].

Diarrhea is perhaps the most often referenced outcome related to water and food-borne diseases. The World Health Organisation [14] found that 2.4% of worldwide diarrhea cases was attributable to climate change in 2000, which would imply about 47,000 additional deaths or 1,459,000 of DALYs in 2000 attributable to climate change [15]. Salmonella and cholera are another two health outcomes from this domain attributable to climate change. Kovats *et al.* [16], for instance, estimate that 35% of all recorded incidences of salmonella, including in Europe, is associated with the effect of temperature on infection transmission, while Watkiss *et al.* [1] note that each degree increase in weekly temperature may increase cases of salmonella by around 5 to 10 per cent. Cholera, another water-borne disease, appears mainly outside Europe. Trærup *et al.* in this volume [5] estimate an increase in the relative risk for cholera cases in Tanzania due to a one degree Celsius increase in temperature by 15 to 29 percent that would imply between 15,000 and 20,000 additional deaths, or about 179,000 and 369,000 additional DALYs for a 2 °C Scenario in 2030.

Climate may also determine the spread of vectors that carry a wide range of diseases, including malaria, dengue fever, tick-borne diseases, or Lyme disease. Most of these health outcomes are potentially significant in developing countries and addressed in global studies, although some of them, such as tick-borne diseases, are highly relevant to Central Europe. WHO [14] estimates that 6% of malaria in some middle-income countries in 2000 was attributed to climate change. McMichael *et al.* [15] provide then a rough estimate of the burden of disease in terms of mortality and DALYs in 2000 attributable to climate change of 27,000 or 1,018 DALYs respectively [4].

Food and water shortages may lead to malnutrition and dehydration that consequently affect the health and involve additional deaths. For instance, McMichael et al [15] estimate malnutrition may cause about 77,000 additional deaths, or almost 3 million DALYs in 2000 that might be attributable to climate change.

Keim [17] and Ebi [18] then assess the relative importance of health effects due to extreme weather events and find that wildfires may cause relatively more fatalities, few to moderate, while other extreme weather events such as storms, floods, and drought cause fewer adverse health impacts, although flash floods may cause quite a large number of them. Again, quoting the McMichael *et al*. study [15], floods may have led to about 2,000 deaths or 193,000 DALYs in 2000 attributable to climate change.

Heat-related health impacts, and particularly heat-related additional deaths, are probably the most important and the most studied health effect attributable to climate change. In fact, the European Environmental Agency [19] identified heat waves as having been the most prominent hazard causing premature mortality over past decades. In a similar vein, recent work as part of the FP7 Climate Cost project [1] shows that heat-related mortality is the key economic impact for health with an order of magnitude impact higher almost than any other health impact considered, and is a clear priority. Most human fatalities caused by heat may be attributable to cardiovascular, cerebrovascular and respiratory causes, primarily among the elderly and poor people living in cities, as reviewed by another paper from this volume [20]. It has been documented that the 2003 summer heat wave resulted in at least 35,000 [1] to 70,000 [21] excess deaths over a few months in Europe, with the daily mortality of the population above 65 years-old increased by 36% in Barcelona, 44% in London, and 105% in France [22]. However, heat is not only a problem of the South; Barriopedro *et al.* [23] estimate an intense heat wave in 2010 led to about 55,000 deaths in Eastern Europe.

There is also increasing evidence for the synergic effect of high temperatures—usually coinciding with dry periods—and air pollution exacerbated through the ozone and more particulate matters that remain in the air during dry periods [21,24,25]. As a matter of fact, climate change mitigating policies may also have an ancillary effect in terms of reduced air quality pollutants and consequently a further positive effect on human health and ecosystems [26,27].

On the other hand, an increase in average temperature will also reduce excess winter deaths and bring benefits. For instance, the PESETA project [1] found the net balance of mortality might even be positive, *i.e.*, the benefits of reduced cold-related mortality in winter are greater than the negative impacts of higher heat-related mortality projected for the summer. This conclusion, however, holds for Europe as a whole and for some model assumptions used in their modeling. Although the benefits from cold-related mortality may balance heat-related mortality in some cases, heat-related health effects may require a policy action. The rationale for action ensues from a fact that the distribution of heat-related and cold-related impacts due to a temperature increase vary significantly across latitudes and, as a consequence, the net balance of premature mortality may be very different across regions. This conclusion is also in line with the World Health Organization that requires detailed assessments of national vulnerabilities to specific health risks to be performed [28].

Moreover, with climate change, the climate change–induced excess health risk as estimated in the recent past, as in the McMichael *et al.* study [15], will be most likely much larger in the near future, as established in the case of diarrhea in developing countries [29], or as predicted for coastal flood risks or due to malnutrition [15].

All these findings show the importance of assessing health risks from climate change, especially excess deaths attributable to climate change. They also emphasize the importance of building a proper policy-relevant assessment model and tools such as cost-benefit analyses embodied by proper monetary values for health benefits.

An integrated assessment of climate change impacts, as performed by means of an Integrated Assessment Model such as FUND [30] or DICE [31], represents probably one of the most comprehensive cost-benefit analyses that utilize monetized human health effects. The FUND model, when valuing the social cost of carbon, considers the health effect of regional temperature on diarrhea, vector-borne diseases such as malaria, schistosomiasis, or dengue fever, and heat-related cardiovascular and respiratory mortality [32]. The corresponding mortality risks are then valued through the value of preventing fatalities, or the value of a statistical life. Contrary to this approach, the second integrated model, DICE [33], relies on estimates based on the global incidence of climate-related disease expressed in terms of years of life lost (YLLs) and DALYs lost as espoused by Murray and Lopez [34]. Identified health effects are divided into climate-related and non-climate related, with the former including dengue fever, malaria and a broad group of tropical diseases. They then use three approaches to estimate the health impact. The first assumes that one-half of the change in LLYs for climate-related diseases is lost as a result of a 2.5 °C warming, while the other approach considers an adjustment to the change in YLLs for each region. Their final method derives the health impact indirectly using the coefficients from regressing the logarithm of climate-related YLLs divided by GDP on the mean regional temperature [33]. In order to monetize these health effects, both of these models consider monetary values from other valuation studies [35,36,37], and assume that the money equivalent of the health impacts depend linearly on a region’s wealth. Specifically, in the FUND model [32], mortality benefits are valued as 200 times per capita income based on Cline [35], while Nordhaus and Boyer [33] assume that a YLL is worth two years of per capita income in their DICE model.

## 3. Valuation of Mortality Risks

### 3.1. What is a VSL

The Value of a Statistical Life (VSL) is a summary measure of the willingness-to-pay for a mortality risk reduction, and a key input into the calculation of the benefits of policies or projects that affect mortality risk or excess death. The mortality benefits are computed as VSL × L, where L is the expected number of excess deaths avoided by the policy.

The VSL is the marginal value of a reduction in the risk of dying, and is therefore defined as the rate at which the people are prepared to trade off income for risk reduction:

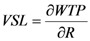
(1)
where R is the risk of dying, and WTP is willingness-to-pay of an individual for reducing the risk by ΔR.

The VSL can equivalently be described as the total willingness to pay by a group of N people experiencing a uniform reduction of 1/N in their risk of dying. To illustrate, consider a group of 10,000 individuals, and assume that each of them is willing to pay €200 to reduce his, or her, own risk of dying by 1 in 10,000. The VSL implied by this WTP is €200/0.0001, or €2 million. The concept of VSL is generally deemed as the appropriate construct for ex ante policy analyses, when the identities of the people whose lives are saved by the policy are not known yet. As shown in the above mentioned example, in practice VSL is computed by first estimating WTP for a specified risk reduction ΔR, and then by dividing WTP by ΔR.

In many countries, including the US and the UK, the VSL used in environmental policy analysis are derived from compensating wage studies [38] or from the literature about transportation accidents. Concerns have been raised about the appropriateness of such practices, because the preferences observed in labor markets are those of workers—not those of the elderly and children, the primary beneficiaries of environmental health protection—and because workplace and transportation risks are very different from the mortality risks associated with environmental exposures [39].

### 3.2. Valuation Methods

The value of preventing fatality, or more often called a value of a statistical life, can be directly derived from people’s preferences to avoid risk of dying revealed in real daily situations [40,41]. However, the revealed preference studies can be used to derive a welfare estimate for only such health-specific non-marketed goods that are embodied in other good traded at market. As an example, preference for occupational risks can be derived from worker’s wage that she accepts at labour market similarly as a value of safety can be derived from market price of safety product such as helmet or seatbelt installed in a vehicle.

In real world, however, it is quite often that there are no marketed goods that can be utilised to estimate welfare measure for many health outcomes. For such cases, preference for health risks can be only elicited under a hypothetical contingent scenario by using one of stated preference valuation method, such as contingent valuation or conjoint choice experiments [42,43]. Using the stated preference technique also allows the valuation can be performed for various contexts, for various beneficiaries, or for various modes of risk reduction delivery.

Nowadays, researchers can benefit from huge number of studies that have examined effect of various contexts or carried out with different designs and treatments. Effect of various characteristics of research design, valuation technique used or different contexts on magnitude of VSL estimates has been already studied in several meta-analyses [40,44,45,46,47,48]. One of the most recent one [49] focused primarily on VSL stated preference studies and utilized over 900 VSL estimates being recorded in OECD database [50] and estimate a VSL mean as high as €4.8 million with median of €3.6 million. Based on benefit transfer then same authors recommend using a range of €0.8 to €8.4 million for a VSL value for OECD countries (all figures recalculated from 2,005 USD into 2,005 Euros).

### 3.3. Our Valuation Method

In this paper, we use discrete choice experiments to obtain an estimate of the VSL. Discrete choice experiments are a survey-based technique used to investigate the tradeoffs that people are prepared to make between different goods or policies [51,52]. It is a stated-preference technique, in that it relies on individuals saying what they would do under hypothetical circumstances, rather than observing actual behaviors in marketplaces [53].

In a typical discrete choice experiment survey, respondents are shown alternative variants of a good or a policy described by a set of attributes, and are asked to choose their most preferred [54]. The alternatives differ from one another in the levels taken by two or more of the attributes. Price is usually one of the attributes, which allows the analyst to estimate the value people ascribe to the good or the monetized benefits of the policy. The choice responses are assumed to be driven by an underlying random utility model. See Alberini, Longo and Veronesi [55] for basic econometric models used with discrete choice experiments.

The choice responses are assumed to be driven by an underlying random utility model. Most applications to date have adopted indirect utility functions that are linear in the attributes and in residual income. Lusk and Norwood [56] study the effects of experiment designs and models in the presence of interactions between attributes, and Alberini *et al.* [57] adopt an indirect utility function that is non-linear in the coefficients and in the attributes. More elaborate models that allow for preference heterogeneity such as mixed logit and latent class models are presented by Swait [58].

One advantage of discrete choice experiments is that they allow the analyst to study people’s responsiveness to goods, levels of environmental quality, or policy offerings that do not currently exist. Another major advantage is that the attributes can be manipulated independently of one another, allowing the analyst to disentangle their effects separately. This is a great advantage when in real life attributes tend to be bundled together. Discrete choice experiments were used to value mortality risk reductions in various contexts [43,57,58,59,60,61,62].

## 4. Research Design and Survey Implementation

### 4.1. Our Discrete Choice Questions

The alternatives in our discrete choice experiments are defined by four attributes: (i) the mortality risk reduction, which is expressed as X in 1,000 over a decade, (ii) latency, *i.e.*, the number of years from now when the risk reduction begins, (iii) whether it’s a blip or a permanent risk reduction, and iv) the cost to the respondent, which will be paid every year starting now and for each of the next 10 years.

We selected a total of four possible risk reductions, ranging from 2 to 5 in 1,000 over 10 years, which are equivalent to 25—in 10,000 per year. Regarding attribute (ii), our blips are risk reductions that last only one decade. Permanent risk reductions take place over the current decade and the next three for respondents aged 40–49 (for a total of four decades), and over the current and two future decades for respondents aged 50–60 (for a total of three decades). By current decade, we mean the one that begins now and lasts for the next 10 years.

The cost amounts were selected to correspond to a wide range of possible VSLs. We chose annual payment for 10 years, rather a one-time payment, because such an extended payment period was judged to be better compatible with the duration of the risk reductions, and because it allowed us to cover a greater range of possible VSL values. Attributes and attribute levels are summarized in Table 1.

**Table 1:** Attributes and attribute levels of the discrete choice experiments.

Risk reduction	2, 3, 4, and 5 in 1,000 per decade
latency	0, 2, 5, 8 years
blip v. permanent	if *blip*, then the risk reductions lasts only for one decade; if *permanent*, the risk reduction lasts 40 years (= 4 decades) if the respondent is aged 40–49 and 30 years (= 3 decades) if the respondent is aged 50–60
cost	annual for the next 10 years, starting this year. The amounts are 4,300, 8,500, 17,000, 30,000, 50,000 CZK
Note: We use purchasing power parity for private consumption of 17.14 Czech crowns to recalculate all values in Euro. Market exchange rate is 25.3 CZK per Euro (2010).	

Our experiment design incorporates a number of restrictions. We wanted our respondents to examine a total of five pairs of hypothetical alternatives. We restricted the latency period to be the same across alternative A and B within a pair, but allowed it to vary across pairs shown to the same respondents, and across respondents. We imposed that (1) the first two pairs of alternatives should be comprised exclusively of blips, (2) in the third pair of alternatives, both alternative A and alternative B should be posit permanent risk reductions, and (3) the last two pairs of alternatives should pitch a blip against a permanent risk reductions.

To create the final experiment design, we first constructed all possible combinations of the attribute levels that complied with the specified restrictions, excluded those with obviously dominated alternatives (or two identical alternatives), and selected at random among the remaining pairs. The resulting design consists of 32 sets (“blocks”) of five pairs of alternatives. Respondents were assigned at random to one of these 32 variants of the questionnaire.

Our study design also included a number of “split sample” treatments. These are described in Alberini *et al.* [42]. Among other things, respondent were randomly assigned to variants of the questionnaire where the risk reductions referred to “all causes of death” cardiovascular and respiratory illnesses, and cancer. One respondent only considered one cause of death in all discrete choice questions.

The discrete choice questions were placed roughly in the middle of the questionnaire. The survey instrument started with eliciting the respondent age, gender and health status, familiarity with cardiovascular illnesses and cancer, and continued with a section that attempted to assess the respondent’s grasp of the notion of life expectancy. We then presented respondents with a simple probability tutorial, which led to the notion of mortality risks. 

As in previous research, we relied on two types of visual aids for conveying risks: grids of squares and bar charts, where the latter are used to show how the risk of dying changes with age. We also told respondents that it is possible to reduce the risk of dying, and that such reductions can be attained through a variety of measures ranging from medical diagnostic tests, car safety equipment, public programs to reduce pollution, *etc.* This was followed by a discussion of the duration of such risk reductions, which is important because one of the attributes of our discrete choice questions is the duration of the mortality risk reduction.

Before asking to choose the most preferred alternative, we introduced two types of mortality risk reductions. The first type occurs for a limited period of time (*i.e.*, the current decade), but in future decades mortality risks will not change. The second type takes places in the current and all future decades. Clearly, these are the description of a “blip” and a permanent mortality risk reduction, although we do not use the word “blip” in our questionnaire. We emphasize that both imply gains in life expectancy, and graphically depict the two types of risk reductions using bar charts.

Respondents were told that in the rest of the questionnaire, we would be focusing on two types of risk reductions: “blips” where the risk reduction occurs over the next 10 years, and permanent risks reductions, where a similar risk reduction takes place in each of the next decades. This is followed by the discrete choice questions, and by debriefing questions which are used to identify any instances of so-called “attribute non-attendance” [63]. The questionnaire concluded with the usual socio-demographic questions.

### 4.2. Survey Implementation

We asked IPSOS Tambor, a well-known survey firm headquartered in Paris, to conduct our surveys in the Czech Republic. We commissioned a sample of N = 2,400 computer-assisted personal interviews (CAPIs), plus an additional sample of computer-assisted web interviews (CAWIs) (N = 800). The CAPI interviews should be geographically representative of the entire country, with oversampling for the five larger cities (Prague, Brno, Ostrava, Plzen and Liberec), so that the 800 completed questionnaires from these cities could be compared with the CAWI sample, which was also to be drawn from the five cities in the Czech Republic. The CAPI interviews were completed at the respondent’s home.

The sample was restricted to respondents aged 40–60. We further requested (1) an even number of men and women, (2) that the educational attainment of the respondents should mirror that of the population in each age group, (3) that each of four age categories reflect respective share in given population, and (4) that 50% of the respondents should have income below the population median household income. The CAWI sample was intended to include people living in large and relatively polluted Czech cities. For purposes of comparison, we decided that out of our total CAPI sample of 2,400 people, 800 would be interviewed in the same cities, so that their responses could be compared to those of the CAWI respondents.

Our instrument was comprehensively pretested during 2009 and 2010. A CAPI pilot was administered on 9–14 July 2010 on a sample of 180 respondents, while the final survey was in the field during 11 August–18 September 2010.

## 5. Data

The CAPI and CAWI samples include respondents from five large cities in the Czech Republic with comparable shares of each of the five cities in both. On the top, the CAPI survey also interviewed 1,577 people living in other than the five cities. Table 2 displays the breakdown of the final sample by mode of administration.

**Table 2:** Description of the sample.

	CAWI		CAPI		ALL	
	N	% of the five cities	N	% of the five cities	N	% out of all
Brno	182	20.3%	186	23.5%	368	11.3%
Liberec	51	5.7%	54	6.8%	105	3.2%
Ostrava	187	20.9%	176	22.3%	363	11.1%
Pilsen	107	12.0%	101	12.8%	208	6.4%
Prague	368	41.1%	273	34.6%	641	19.7%
**Total five cities**	**895**		**790**		**1** **,** **685**	
Other municipalities	NA		1,577	67%	1,577	48.3%
**Total**	**895**		**2** **,** **367**		**3** **,** **262**	

Table 3 reports descriptive statistics for some variables for the two samples defined by the mode of survey administration when we consider only observations from the respondents living in the five larger Czech cities. The mean age of entire sample was 49.4 years and the means for the CAWI and CAPI samples are not statistically different. Males and females are evenly represented in our two samples, although there are slightly more males in the CAPI sample. The two samples also differ with respect to household structure, income and education. There are more married in CAPI, 75% *versus* 68% of the respondents. The CAPI respondents have also more young children (below 18) and less older children than 18. The mean of net income per family person is about 13,000 CZK a month (about €750 by purchasing power parity) and is larger for the CAWI respondents, while the mean of net household income is then 22,000 and 23,000 CZK a month (about €1,300) and is slightly larger for the CAPI respondents.

**Table 3:** Descriptive statistics of the CAWI and CAPI five-city samples.

Variable name	Unit	CAWI (N = 895)		CAPI (N = 790)		Equality of sample means
		Mean	Std Dev	Mean	Std Dev	t stat
age	years	49.53	6.26	49.22	6.37	1.01
male	%	0.44	0.50	0.50	0.50	−2.42
married	%	0.68	0.47	0.75	0.44	−3.06
household size	number	2.78	1.16	2.75	1.12	0.55
children	number	2.04	2.29	1.88	1.39	1.75
children (younger 18)	number	0.60	0.84	0.68	0.91	−1.71
income (personal)	CZK a month	13,609	7,435	12,863	6,827	1.87
income (household)	CZK a month	22,040	10,142	22,929	10,162	−1.79
education: basic	[dummy], %	0.09	0.28	0.17	0.38	−5.31
education: secondary	[dummy], %	0.29	0.45	0.36	0.48	−3.18
education: A level	[dummy], %	0.41	0.49	0.27	0.44	6.31
education: bachelor	[dummy], %	0.05	0.22	0.04	0.20	0.81
education: university	[dummy], %	0.14	0.34	0.15	0.35	−0.61

There are more respondents interviewed via the internet with A-level decree secondary education and less with basic and secondary education without A-level. Shares of respondents with bachelor or higher university decree are about 5% and 15% and same for the two samples.

Preference for health risks may be affected by respondent’s own health status, perception about own health and life expectancy and aversion to health risks. To get known who our respondents are we asked the respondents to assess their own health (by using 5-point Lickert scale: excellent-very good-good-fair-bad), and what age they most likely will live (a scale with 5-years intervals starting from the respondent’s age), and whether there are or were smokers. Table 4 summarizes statistics for the four questions. Most of our respondents, about 60%, perceive their own health good and better and all the shares do not significantly differ across the two samples. There are, however, slightly more respondents interviewed in person who judge their own health bad. Most of the respondents think they will live until their 76 and 85 years; after some recalculations we found the mean of expected survival at about 81.6 years. One’s lifetime as subjectively perceived is then not statistically different across the two samples. Percentages of recent or past smokers are same across the samples.

**Table 4:** Self-assessment of own health and age until when one most likely will survive.

Variable name	Unit	CAWI-5cities (N = 895)		CAPI-5cities (N = 790)		Equality of sample means
		Mean	Std Dev	Mean	Std Dev	t stat
health excellent	[dummy], %	0.05	0.21	0.04	0.20	0.49
health very good	[dummy], %	0.14	0.35	0.14	0.34	0.51
health good	[dummy], %	0.43	0.50	0.42	0.49	0.26
health fair	[dummy], %	0.29	0.45	0.28	0.45	0.22
health bad	[dummy], %	0.09	0.29	0.12	0.32	−1.74
smoker	[dummy], %	0.37	0.48	0.40	0.49	−1.42
smoker former	[dummy], %	0.25	0.44	0.22	0.42	1.54
expected one’s lifetime	10-point category (in years of age)	5.34 (81.68)	1.53 (8.12)	5.35 (81.61)	1.56 (8.49)	−0.08 (0.16)

## 6. Model and Results

We base our estimates on the random utility model, assuming that the indirect utility has the following form:

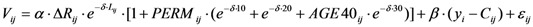
(2)
where α is the marginal utility of a unit risk reduction, ΔR is the risk reduction per year, L is latency, *i.e.*, the number of years from now when the risk reduction begins, δ is the discount rate, β is the marginal utility of income, y is income and C is the payment that must be incurred in the first year, and subscripts *i* and *j* denote the respondent and the alternative, respectively. PERM is a dummy that takes on a value of 1 if the alternative reduces the mortality risk permanently, otherwise it is equal to zero when the risk reduction only lasts for 10 years (“blip”). In the case of a permanent risk reduction, the risk reduction lasts for four consequent decades for a respondent between 40 and 49 years, or for three decades when a respondent is 50−60 years old. To distinguish this in our model, a dummy variable AGE40 takes a value of one when a respondent is 40−49 years old. The coefficients α, β, and δ need to be estimated.

The term ε is an independent and identically distributed type I extreme value error term with a scale parameter equal to 1, and the probability that respondent *i* choses alternative *k* is:

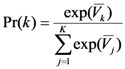
(3)


This means that the appropriate statistical model of the responses is a conditional logit that is non-linear in the parameters, and the probability is Equation (3) is the contribution to the likelihood of the conditional logit model.

The VSL equals the marginal utility of a unit risk reduction weighted by the marginal utility of income:

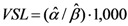
(4)


Because in our estimation we express the risk reduction as, say, 2 or 3 (in 1,000), instead of 0.002 or 0.003, we multiply the ratio by 1,000.

All estimates of the VSL are reported in millions of Euros expressed by the purchasing power parity of 17.14 CZK per Euro. The standard errors around the estimates of the VSL are computed using the delta method. For simplicity, in this paper the analysis is limited to the responses to the first discrete choice question for each choice task (*i.e.*, which is your most preferred out of A, B, or the *status quo*?). We remind the reader that VSL estimates should be interpreted as the average of the VSL for all causes of death, cardiovascular and respiratory illnesses, and cancer deaths that are the three causes we used in our discrete choice experiments.

At first, Table 5 reports estimates of the VSL for four different samples: CAPI + CAWI denotes the entire sample of all respondents (N = 3,262 respondents) interviewed both on-line and in-person; the CAPI sample includes all observations made from in-person interviews of respondents from the whole Czech Republic, including the five larger cities (N = 2,367), while CAPI-5cities includes only respondents from the five larger cities (N = 790); finally, CAWI-5cities includes respondents from the e-panel interviewed on-line and who are all living in the five larger cities (N = 895).

**Table 5:** Results for different samples of respondents, conditional logit model.

	CAPI + CAWI		CAPI		CAPI-5cities		CAWI-5cities	
	*coeff.*	*s.e.*	*coeff.*	*s.e.*	*coeff.*	*s.e.*	*coeff.*	*s.e.*
*α*	1.03	0.0902	1.06	1.06	1.39	0.178	0.96	0.173
*β*	−0.00043	1.39E-05	−0.00043	−0.00043	−0.00055	2.94E-05	−0.00043	2.68E-05
*δ*	0.0866	0.00988	0.085	0.085	0.0894	0.014	0.0913	0.0214
N of observations	16,310		11,835		3,950		4,475	
VSL (mill euro)	2.41		2.48		2.55		2.25	

The results reported in Table 5 confirm that the responses to the discrete choice questions are consistent with the economic paradigm. The marginal value of the risk reductions is always positive and significant, and the marginal utility of income (*i.e.*, the negative of the coefficient on cost, which is what we display in all the tables below) positive and significant. The VSLs for all four samples range between 38 and 44 million Czech crown, or 2.25 and 2.55 million euro respectively, and neither one is statistically different form all the others at any convenient level.

All coefficients, *i.e.*, the marginal utilities of a risk reduction (*α*), the marginal utility of income (*β*), and implicit discount rate (*δ*), are virtually the same for the sample of respondents interviewed in-person (CAPI sample) and for the e-panel (CAWI-5cities) (with Wald test statistics of 0.243, 0.001, and 0.068 respectively). However, the two samples are not fully comparable due to having respondents from smaller cities and towns in the CAPI sample.

We therefore compare the coefficients for the two comparable samples, CAPI-5cities and CAWI-5cities and found same coefficients for the marginal utility of a risk reduction and implicit discount, but not for the marginal utility of income (Wald = 8.95, *p*-value = 0.003). We note that we even followed the same sampling strategy while recruiting people to both our 5-city samples (see Section 4, Survey Implementation), and these two samples slightly differ with respect to several main socio-demographic characteristics, including income (see Table 3 and Table 4). Overall, expressed in PPP euro, the VSL is €2.25 million in the CAWI-5cities sample, and €2.55 million in the CAPI-5cities sample, and these two figures are not statistically different from one another.

Further, we investigate whether VSL estimates differ between the two modes of survey administration in Prague, the capital of the Czech Republic, and between Prague and the other four large Czech cities where the survey was carried out by the two different modes of administration.

Interestingly, we find the mode of survey administration—CAWI or CAPI—does not significantly affect the VSL as estimated for Prague, or for the remaining four larger Czech cities as shown in Table 6. Despite the fact that the VSLs are not statistically different across the mode of survey administration, both marginal utilities of a risk reduction and of income differ for Prague respondents between the two survey modes (Wald statistics of 6.03 and 13.90). The two marginal utilities are not, however, statistically different between the two modes for the respondents living in the four large cities (Wald 0.75 and 0.38). Respondents from Prague and those living in the four large cities exhibit different marginal utilities of a risk reduction and discount rates, which implies different magnitudes of VSLs when the survey modes are same. This shows in different respondents’ preference structure and the socio-demographic characteristics of the two sub-samples.

**Table 6:** VSL estimations for different cities and the mode of survey administration.

	Prague				4 cities			
	CAPI		CAWI		CAPI		CAWI	
	*coeff.*	*s.e.*	*coeff.*	*s.e.*	*coeff.*	*s.e.*	*coeff.*	*s.e.*
*α*	2.26	0.312	1.25	0.268	0.97	0.217	0.70	0.222
*β*	−0.00056	4.95E-05	−0.00033	3.99E-05	−0.00054	3.68E-05	−0.00051	3.64E-05
*δ*	0.095	0.0158	0.123	0.0312	0.083	0.0232	0.055	0.0256
N of obs.	1,365		1,840		2,585		2,635	
VSL (mill Euro)	4.02		3.85		1.79		1.37	

Lastly, we want to investigate whether any differences in the VSL and in the coefficients of the indirect utility appear between respondents in their forties and the respondents in their fifties. Interestingly enough, we find again the VSLs are not statistically different between the modes of survey administration at any convenient level (Table 7). However, in the case of older respondents, the marginal utility of a risk reduction and the marginal utility of income are both much larger for the respondents interviewed on-line and included in the internet panel (Wald statistics are 7.06 and 17.24, resulting in *p*-values of 0.008 and <0.000). This finding shows at quite different preferences of older people who participate in the internet panels and thus may have quite different lifestyle than the ones who do not participate in the e-panels or even not use the internet at all.

**Table 7:** VSL estimations for different age of respondents and the mode of survey administration.

	age 40−50				age 51−60			
	CAPI		CAWI		CAPI		CAWI	
	*coeff.*	*s.e.*	*coeff.*	*s.e.*	*coeff.*	*s.e.*	*coeff.*	*s.e.*
*α*	1.16	0.232	1.17	0.235	1.70	0.275	0.71	0.254
*β*	−0.00050	0.000	−0.00048	0.000	−0.00062	0.000	−0.00036	0.000
*δ*	0.076	0.017	0.087	0.021	0.108	0.021	0.107	0.049
N of obs.	2,220		2,495		1,730		1,980	
VSL (mill euro)	2.33		2.44		2.76		1.94	

The discount rate is about 9% in all the samples presented in Table 5. This suggests that future risk reductions are actually greatly discounted by our respondents. This result is in sharp contrast with earlier studies [14,43], where we found that the discount rates were virtually zero, but it is close to another study conducted in Italy where respondents actually discount future risk reductions at a similar rate (about 7%) [57]. Respondents from Prague are slightly less patient than the respondents from the other four cities. Moreover, it seems that the implicit discounts are slightly larger for the respondents interviewed on-line than in-person in Prague (compare 12.3% *versus* 9.5%). Conversely, larger discounts are indicated by those interviewed in-person in the other four cities (8.3% *versus* 5.5%). Nevertheless, neither of these coefficients are statistically different from one other. In line with one’s expectations, we also found that our older respondents were less patient than our respondents in their forties, selecting a discount rate of about 11% compared to rates of about 8 to 9 percent. In summary, the mode of survey administration does not have any significant effect on the estimate of implicit discount rate.

Can be the value of preventing a fatality, as used in some integrated assessment models that quantify cost of climate change, be justified? In brief, yes it can. In the FUND model, mortality benefits are valued at 200 times per capita income [9]. Considering the per capita disposable income in the Czech Republic in 2010 (that is about €9,700 in purchasing power parity), it implies a value of a human fatality for the Czech Republic, as used in the FUND model, of about €1.95 million, which is a value very close to our estimate. In the DICE model, health impacts are valued through the Year of Life Lost measure that is worth two years of per capita income. In this case, the Czech value of YLL would equate to a magnitude slightly less than €20,000. Although both these measures, *i.e.*, statistical life and YLL, are indicators of premature mortality, these two are not directly comparable; however, as a rough approximation, we found the value of health impact, as used in the DICE model, is about three times smaller than the value would be if it was based on our estimate of VSL and used for an average person in the Czech population [64]. The monetary value of YLL can, however, be directly compared to the estimate of VOLY. For instance, the NEEDS study derives a median value of VOLY for the Czech sample of about €19,000 (for a 3-month life expectancy gain), or €12,400 (for a 6-month gain), respectively [65]. Using the same study but mean values, Desaigues *et al.* [65] then recommend using a VOLY of €33,000 for New Member countries, *i.e.*, those that became an EU member in 2004 and later. 

Bearing in mind the differences in valuation method, the design of the valuation tool, the target population, and the sampling plan, our result is comparable to other estimates derived from the preferences of individuals from the same geographical region. For instance, a contingent valuation study [66] elicited a willingness to pay from respondents living in the three largest Czech cities in 2004 for reducing the risk of dying from cardiovascular and respiratory diseases and a derived median VSL of €2.55 million. Alberini and Ščasný [43] estimate a VSL of €1.08 million through using discrete choice experiments in a sample of Czech parents in 2008. VSL is also comparable to two hedonic wage studies; a VSL estimate in Poland ranges from €1.6 million to €3.3 million, depending on the level of data disaggregation to define industry dummies [67], while a VSL derived for Czech employees lies somewhere between €10 million and €16 million if an objective measure of risk is used, or about €3.5 million if a subjective perception of occupational risk is used instead [41,68,69].

## 7. Conclusions

We developed a survey questionnaire specifically designed to examine several hypotheses about mortality risk reductions and administered them to independent samples of respondents in the Czech Republic. One sample was interviewed via CAPI, in person, and the other via CAWI, via the internet and using an internet panel.

The valuation task consisted in a series of discrete choice experiments. Each person was asked to examine five pairs of hypothetical risk reduction profiles, and for each pair, the respondent was asked to choose the most preferred. Each profile was defined by four attributes: the size of the risk reduction, whether the risk reduction is effective for this decade only or is repeated, whether it starts right away or is delayed, and finally the cost, to be paid annually for each decade.

The responses to the discrete choice questions were reasonable and are consistent with the economic paradigm, as were the VSL values. Expressed in PPP euro, the VSL is about EUR 2.4 million, or EUR 1.65 million using the 2010 exchange rate [70]. Our estimate of a VSL for the Czech Republic is also pretty much same as the value of preventing a fatality used in the FUND model.

If we differentiate the mode of survey administration, we find in the five Czech cities a VSL of €2.25 million in the CAWI sample and €2.55 in the CAPI sample. Importantly, these figures are not statistically different from one another. As Lindhejm and Navrud [13] found, the mode of survey administration does not have a significant effect on the VSL estimate, although the magnitude of the VSL for the internet survey is slightly smaller.

We found the future risk reductions are greatly discounted by our respondents, on average, with an implicit discount rate of about 9%, and we found that neither of the coefficients for the discount rate is statistically different one from the other. We therefore conclude that the mode of survey administration does not have any significant effect on the estimate of implicit discount rate.

However, it seems that the mode of administration does have an effect on other key parameters of indirect utility. In particular, the marginal utility of a risk reduction differs between the two modes of administration for respondents from Prague, and the marginal utility of income is different between the modes, especially in the case of older respondents (over 50 years old) and among respondents from Prague. This is demonstrated in the different preferences of older people in particular who may have participated in the internet panels compared to those in their fifties who did not participate in the e-panels or who do not necessarily use the internet at all. We also detected a different preference pattern for respondents living in large agglomerations compared to smaller Czech cities. This observation emphasizes the need to perform proper sampling to maintain the representation of the CAPI and CAWI samples taken from specific segments of populations, such as those living in very large cities or older people.

We conclude that internet surveys are a reasonable way of administering a complicated questionnaire on cognitively demanding topics, such as mortality risk reductions. However, the differences in our estimates of the key parameters of the indirect utility for very specific segments, such as the population from very large cities or older people, suggest we should pay attention to representation of internet panels and internet samples. A mixed-mode of survey administration that utilises both of the survey modes might improve representation of the whole sample, especially in the case of the specific segments of population interviewed and particularly in countries with rather low internet penetration.

## References

[1] Watkiss P., Horrocks L., Pye S., Searl A., Hunt A. (2009). Impacts of Climate Change in Human Health in Europe. PESETA—Human Health Study.

[2] Campbell-Lendrum D., Woodruff R., Prüss-Üstün A., Corvalán C. (2007). Climate Change: Quantifying the Health Impact at National and Local Levels.

[3] Intergovernmental Panel on Climate Change. Working Group II (2007). Climate Change 2007: Impacts, Adaption and Vulnerability.

[4] Organisation for Economic Co-operation and Development (2008). Costs of Inaction on Key Environmental Challenges.

[5] Trærup S.L.M., Ortiz R.A., Markandya A. (2011). The costs of climate change: A study of Cholera in Tanzania. Int. J. Environ. Res. Public Health.

[6] Desaigues B., Ami D., Bartczak A., Braun-Kohlová M., Chilton S., Czajkowski M., Farreras V., Hunt A., Hutchison M., Jeanrenaud C., Kaderjak P., Máca V., Markiewicz O., Markowska A., Metcalf H., Navrud S., Nielsen J.S., Ortiz R., Pellegrini S., Rabl A., Riera R., Scasny M., Stoeckel M.-E., Szántó R., Urban J. (2011). Economic valuation of air pollution mortality: A 9-country contingent valuation survey of value of a life year (VOLY). Ecol. Indic..

[7] Berrens R.P., Bohara A.K., Jenkins-Smith H.C., Silva C.L., Weimer D.L. (2004). Information and effort in contingent valuation surveys: Application to global climate change using national internet samples. J. Environ. Econ. Manage..

[8] Alberini A., Cropper M., Krupnick A., Simon N.B. (2004). Does the value of a statistical life vary with age and health status? Evidence from the US and Canada. J. Environ. Econ. Manage..

[9] Alberini A., Cropper M., Krupnick A., Simon N.B. (2006). Willingness to pay for mortality risk reductions: Does latency matter?. J. Risk Uncertainty.

[10] Adamowicz W., Dupont D., Krupnick A., Zhang J. (2001). Valuation of cancer and microbial disease risk reductions in municipal drinking water: An analysis of risk context using multiple valuation methods. J. Environ. Econ. Manage..

[11] Bosworth R., Cameron T.A., DeShazo J.R. (2009). Demand for environmental policies to improve health: Evaluating community-level policy scenarios. J. Environ. Econ. Manage..

[12] Bulte E., Gerking S., List J.A., de Zeeuw A. (2005). The effect of varying the causes of environmental problems on stated WTP values: Evidence from a field study. J. Environ. Econ. Manage..

[13] Lindhjem H., Navrud S. (2011). Using Internet in stated preference surveys: A review and comparison of survey modes. Int. Rev. Environ. Resour. Econ..

[14] World Health Organization (2003). Coastal and Fresh Waters. Guidelines for Safe Recreational Water Environments.

[15] McMichael A.J., Campbell-Lendrum D., Kovats S., Edwards S., Wilkinson P., Wilson T., Nicholls R., Hales S., Tanser F., le Sueur D., Schlesinger M., Andronova N., Ezzati M., Lopez A., Roders A., Murray C.J.L. (2004). Global Climate Change. Comparative Quantification of Health Risks, Global and Regional Burden of Disease Attributable to Selected Major Risk Factors.

[16] Kovats R.S., Edwards S.J., Hajat S., Armstrong B.G., Ebi K.L., Menne B. (2004). The effect of temperature on food poisoning: A time-series analysis of salmonellosis in ten European countries. Epidemiol. Infect..

[17] Keim M.E. (2008). Building human resilience: The role of public health preparedness and response as an adaptation to climate change. Amer. J. Prev. Med..

[18] Ebi K.L. (2011). Resilience to the health risks of extreme weather events in a changing climate in the United States. Int. J. Environ. Res. Public Health.

[19] European Environment Agency (2010). The European Environment: State and Outlook 2010: Air Pollution.

[20] Alberini A., Gans W., Alhassan M. (2011). Individual and public-program adaptation: Coping with heat waves in five cities in Canada. Int. J. Environ. Res. Public Health.

[21] European Environment Agency (2012). Urban Adaptation to Climate Change in Europe: Challenges And Opportunities for Cities together with Supportive National and European Policies.

[22] D’Ippoliti D., Michelozzi P., Marino C., de’Donato F., Menne B., Katsouyanni K., Kirchmayer U., Analitis A., Medina-Ramón M., Paldy A., Atkinson R., Kovats S., Bisanti L., Schneider A., Lefranc A., Iñiguez C., Perucci C.A. (2010). The impact of heat waves on mortality in 9 European cities: Results from the EuroHEAT project. Environ. Health.

[23] Barriopedro D., Fischer E.M., Luterbacher J., Trigo R.M., Garcia-Herrera R. (2011). The Hot summer of 2010: Redrawing the temperature record map of Europe. Science.

[24] Bell M.L., Dominici F., Samet J.M. (2005). A meta-analysis of time-series studies of ozone and mortality with comparison to the national morbidity, mortality, and air pollution study. Epidemiology.

[25] Medina-Ramón M., Zanobetti A., Cavanagh D.P., Schwartz J. (2006). Extreme temperatures and mortality: Assessing effect modification by personal characteristics and specific cause of death in a multi-city case-only analysis. Environ. Health Perspect..

[26] Holland M., Amann M., Heyes C., Rafaj P., Schöpp W., Hunt A., Watkiss P. (2011). The Reduction in Air Quality Impacts and Associated Economic Benefits of Mitigation Policy: Summary of Results from the EC RTD ClimateCost Project. Technical Policy Briefing Note 6: Ancillary Air Quality Benefits.

[27] Kiulia O., Markandya A., Ščasný M., Menkyna Tsuchimoto F. The economic and environmental effects of taxing air pollutants and CO_2_: Lessons from a study of the Czech Republic. Rev. Environ. Econ. Policy.

[28] World Health Organization (2002). The World Health Report 2002.

[29] Patz J.A., Campbell-Lendrum D., Holloway T., Foley J.A. (2005). Impact of regional climate change on human health. Nature.

[30] Tol R.S.J. (2009). The economic effects of climate change. J. Econ. Perspect..

[31] Nordhaus W.D. (2011). Estimates of the social cost of carbon: Background and results from the rice—2011 model. SSRN Electron. J..

[32] Anthoff D., Toll S.J.R. Climate Framework for Uncertainty, Negotiation and Distribution: Technical Description, Version 3.5. 2010. www.fund-model.org/versions.

[33] Nordhaus W.D., Boyer J. (2000). Warming the World: Economic Models of Global Warming.

[34] Murray C.J.L., Lopez A.D. (1996). The Global Burden of Disease.

[35] Cline W.R. (1992). The Economics of Global Warming.

[36] Navrud S. (2001). Valuing health impacts from air pollution in Europe. Environ. Resour. Econ..

[37] Tolley G.S., Kenkel D.S., Fabian R.G. (1994). Valuing Health for Policy: An Economic Approach.

[38] Viscusi W.K. (1993). The value of risks to life and health. J. Econ. Lit..

[39] Robinson L.A. (2007). Policy monitor: How US government agencies value mortality risk reductions. Rev. Environ. Econ. Policy.

[40] Viscusi W.K., Aldy J.E. (2003). The value of a statistical life: A critical review of market estimates throughout the world. J. Risk Uncertainty.

[41] Melichar J., Ščasný M., Urban J. (2010). Hodnocení smrtelných rizik na trhu práce: Studie hédonické mzdy pro Českou Republiku. (The valuation of risks in the labour market: Hedonic wage study in Czech Republic). Politická ekonomie.

[42] Alberini A., Bateman I., Loomes G. (2010). Valuation of Environment—Related Health Risks for Children.

[43] Alberini A., Ščasný M. (2011). Context and the VSL: Evidence from a stated preference study in Italy and the Czech Republic. Environ. Resour. Econ..

[44] Viscusi W.K. (1992). Fatal Tradeoffs: Public and Private Responsibilities for Risk.

[45] Kochi I., Hubbell B., Kramer R. (2006). An empirical Bayes approach to combining and comparing estimates of the value of a statistical life for environmental policy analysis. Environ. Resour. Econ..

[46] Mrozek J.R., Taylor L.O. (2002). What determines the value of life? A meta-analysis. J. Policy Anal. Manage..

[47] de Blaeij A., Florax R.J.G.M., Rietveld P., Verhoef E. (2003). The value of statistical life in road safety: A meta-analysis. Accid. Anal. Prev..

[48] Dekker T., Brouwer R., Hofkes M., Moeltner K. (2011). The effect of risk context on the value of a statistical life: A bayesian meta-model. Environ. Resour. Econ..

[49] Lindhjem H.V., Navrud S., Braathen N.A. (2010). Meta-Analysis of Stated Preference VSL Studies: Further Model Sensitivity and Benefit Transfer Issues. PIMAVE Technical Report.

[50] Braathen N.A., Lindhjem H., Navrud S. (2010). Valuing Lives Saved from Environmental, Transport and Health Policies: A Meta-Analysis of Stated Preference Studies.

[51] Bateman I.J., Carson R.T., Day B., Hanemann M., Hanley N., Hett T., Jones-Lee M., Loomes G., Mourato S., Özdemiroglu E., Pearce D.W., Sugden R., Swanson J. (2002). Economic Valuation with Stated Preference Techniques: A Manual.

[52] Carson R.T., Louviere J.J. (2011). A common nomenclature for stated preference elicitation approaches. Environ. Resour. Econ..

[53] Alriksson S., Oberg T. (2008). Conjoint analysis for environmental evaluation—A review of methods and applications. Environ. Sci. Pollut. Res. Int..

[54] Hanley N., Mourato S., Wright R.E. (2001). Choice modelling approaches: A superior alternative for environmental valuation?. J. Econ. Surv..

[55] Alberini A., Longo A., Veronesi M., Kanninen B. (2007). Basic Statistical Models for Conjoint Choice Experiments. Valuing Environmental Amenities using Choice Experiments: A Common Sense Guide to Theory and Practice.

[56] Lusk J.L., Norwood F.B. (2005). Effect of experimental design on choice-based conjoint valuation estimates. Amer. J. Agr. Econ..

[57] Alberini A., Tonin S., Turvani M., Chiabai A. (2007). Paying for permanence: Public preferences for contaminated site cleanup. J. Risk Uncertainty.

[58] Swait J., Kanninen B. (2007). Advanced Choice Models. Valuing Environmental Amenities using Choice Experiments: A Common Sense Guide to Theory and Practice.

[59] Tsuge T., Kishimoto A., Takeuchi K. (2005). A choice experiment approach to the valuation of mortality. J. Risk Uncertainty.

[60] Itaoka K., Saito A., Krupnick A., Adamowicz W., Taniguchi T. (2006). The effect of risk characteristics on the willingness to pay for mortality risk reductions from electric power generation. Environ. Resour. Econ..

[61] Alberini A., Ščasný M., Guignet D., Tonin S. (2012). Cancer values of prevented fatalities (VPFs), one size does not fit all: The benefits of contaminated site cleanups in Italy. J. AirWaste Manage. Assoc..

[62] Tonin S., Alberini A., Turvani M. (2009). The Value of Reducing cancer Risks at Contaminated Sites: Are More Heavily Exposed People Willing to Pay More?.

[63] Campbell D., Hutchinson W.G., Scarpa R. (2008). Incorporating discontinuous preferences into the analysis of discrete choice experiments. Environ. Resour. Econ..

[64] Moore M.J., Viscusi W.K. (1988). The quantity-adjusted value of life. Econ. Inq..

[B65-ijerph-09-04760] 65.Statistical life is an aggregate of tiny risk reductions in a certain population; for instance, if the risk reduction is reduced by 1 in 1,000 for each person in a population of 1,000 people, then a given program would yield one statistical life overall. The Year of Life Lost is calculated by multiplying the number of deaths by a standard life expectancy at the age at which death occurs. It is recommended Value of Statistical Life Year, VSLY, be used when a certain program results in a difference in longevity. VSLY can be derived from the VSL as “quantity-adjusted value of life” by dividing the VSL estimate by the discounted expected remaining years of life, as (r × VSL)/[1 − (1 + r) − LE], where r denotes the discount rate and LE is the individual’s remaining life expectancy. For instance, for a person with 40 years of remaining life expectancy, assuming a 3% discount rate, the VSLY would be about 4.3% of the VLS value (or 3% of the VSL if one assumes a 1% discount rate). Using the Czech VSL estimate, the VSLY for this person would be worth €104,000 (or €73,000 respectively), which is a five (or three-and-half) times larger value of health impact than has been used in the DICE model

[66] Desaigues B., Ami D., Hutchison M., Rabl A., Chilton S., Metcalf H., Hunt A., Ortiz R., Navrud S., Kaderjak P., Szántó R., Seested Nielsen J., Jeanrenaud C., Pellegrini S.,  Braun Kohlová M., Ščasný M., Vojtěch M., Urban J., Stoeckel M., Bartczak A., Markiewicz O., Riera P., Farreras V. (2007). Deliverable D6.7—Final Report on the Monetary Valuation of Mortality and Morbidity Risks from Air Pollution. Sixth Framework Programme of DG Research European Commission (Project No 502687 “New Energy Externalities Developments for Sustainability” [NEEDS]).

[67] Alberini A., Ščasný M., Braun Kohlová M., Melichar J., Menne B., Ebi K.L. (2006). The Value of a Statistical Life in the Czech Republic: Evidence from a Contingent Valuation Study. Climate Change Adaptation Strategies for Europe.

[68] Giergiczny M. (2008). Value of a statistical life—The case of Poland. Environ. Resour. Econ..

[69] asný M., Urban J., Ščasný M., Braun Kohlová M. (2008). Application of the Hedonic Wage Model: Value of Statistical Life Derived from Employee’s Choice in the Czech Labor Marke. Modelling of Consumer Behaviour and Wealth Distribution.

[B70-ijerph-09-04760] 70.All VSL values are recalculated in 2010 Euro by purchasing power parity

